# Vitamin B12 Deficiency as a Cause of Neurotrophic Keratopathy

**DOI:** 10.2174/1874364101712010007

**Published:** 2018-02-28

**Authors:** Nader Nassiri, Farhad Assarzadegan, Mansoor Shahriari, Hamid Norouzi, Sara Kavousnezhad, Nariman Nassiri, Kourosh Sheibani

**Affiliations:** 1 Department of Ophthalmology, Imam Hossein Medical Center, Shahid Beheshti University of Medical Sciences, Tehran, Iran; 2 Ophthalmic Research Center, Shahid Beheshti University of Medical Sciences, Tehran, Iran; 3 Department of Neurology, Shahid Beheshti University of Medical Sciences, Tehran, Iran; 4 Jules Stein Eye Institute, University of California at Los Angeles, Los Angeles, USA; 5 Basir Eye Health Research Center, Basir Eye Clinic, Tehran, Iran.

**Keywords:** Keratopathy, Deficiency, Vitamin, Neurotrophic, Vitamin B12, Corneal Sensation

## Abstract

**Introduction:**

Neurotrophic keratitis is a rare degenerative corneal disease caused by an impairment of trigeminal corneal innervation, leading to a decrease or absence of corneal sensation. Here, we present a case of neurotrophic keratopathy caused by B12 deficiency in a 34 years old man who had a progressive decrease in visual acuity and corneal involvement since 3 months before being referred to our ophthalmology clinic.

**Result and Discussion:**

Based on our clinical findings and with the diagnosis of B12 deficiency we started B12 treatment for the patient. After 3 weeks the patient showed a dramatic response with corneal sensation reversal, an increase of visual acuity, improved neurotrophic keratopathy and significantly improved neurological findings. To the best of our knowledge, there is no report regarding vitamin B12 deficiency induced keratopathy and this is the first report that describes this aspect of vitamin B12 deficiency.

## 1 INTRODUCTION

 Many different ocular and systemic diseases might cause a lesion at different levels of the fifth cranial nerve leading to decreased corneal sensation. These levels include the nucleus in the pons, the Gasserian ganglion, the trigeminal ophthalmic branch, the nasociliary nerve, or the long ciliary nerve [1].

 The most common causes of corneal anesthesia are viral infections, chemical burns, physical injuries, and corneal surgery [1]. Intracranial space-occupying lesions including neuroma, meningioma, and aneurysms may cause compression of the trigeminal nerve or ganglion leading to reduced corneal sensitivity [2]. Also, many systemic conditions including diabetes, multiple sclerosis, congenital syndromes, and leprosy are associated with decreased sensory nerve function or damage to sensory fibers leading to corneal anesthesia [1].

 A rare cause of this sensation loss is neurotrophic keratitis: a degenerative corneal disease caused by an impairment of trigeminal corneal innervation which leads to a decrease or absence of corneal sensation [3]. The corneal epithelium is the first target of the disease leading to dystrophic changes and defects with a poor tendency to spontaneous healing and the progression of the disease may lead to corneal ulcers, melting and perforation. Conjunctival changes include a decrease of goblet cell density and loss of cell-surface microplicae [1].

 It is relatively easy to diagnose this condition based on patients’ history and clinical findings but its management is very hard and challenging [1]. Here, we present a case of neurotrophic keratopathy caused by B12 deficiency in a 34

years old man who had progressive decrease in visual acuity and eye redness since 3 months before being referred to our ophthalmology clinic.

## 2 CASE REPORT

Here we present a case of neurotrophic keratopathy caused by B12 deficiency. The patient was a 34 year old man, who had started a tight vegetarian diet since 20 months before being referred to our clinic and had progressive decrease in visual acuity and eye redness since 3 months ago. Other clinical findings of our patient were: ataxia, limbal parasthesia and lethargy which were progressive and started 3 months before the referral.

 In slit lamp exam, corneal epithelial defect and mild sub epithelial infiltration were found Fig. (**1**). Corneal sensation was measured using Cochet–Bonnet esthesiometer (Luneau Ophthalmology, Paris, France) which indicated a decrease of corneal sensation. Although, corneal epithelial defect and abrasion were observed after fluorescein staining Fig. (**2**), he did not have any ocular pain, foreign body sensation, and photophobia, and his eyes were inappropriately quiet due to corneal sensation loss. In fundoscopy exam, optic disc was pale in the temporal zone but it had sharp margin and cup/disc ratio was approximately 0.3. Because of corneal epithelium defect, we did not perform Goldman applanation tonometry. After amelioration of corneal epithelium defect, we checked the intraocular pressure and it was normal. The tear meniscus was normal and he did not have an apparent dry eye; we performed Schirmer test for him and it was nearly normal (14mm). Other ocular examinations were normal.

 Neurological examination findings were: ataxia, decrease of vibration and position sensation and limbus parasthesia. Our assessment of patient’s ocular findings was neurotrophic keratopathy and optic neuropathy. The patient underwent paraclinical tests like peripapillary OCT, macular OCT, visual field assessment, lumbar puncture, brain and spinal MRI, lab tests like, CBC, ESR, CRP, vitamins blood levels, homocysteine, and virology tests.

 Positive points of our evaluation were: hyper intensity of posterior column in spinal MRI Fig. (**3**), hyper homocysteinemia (52 micromol/lit, Normal range < 15 micromol/lit), B12 deficiency (pretreatment level 195pgr/ml, Normal range 200-900 pgr/ml), Iron deficiency and normocytic anemia. Peripapillary OCT showed temporal atrophy of optic disc Fig. (**4a,b**). Perimetry also showed a small paracentral scotoma in the patient’s visual field Fig. (**5a,b**).

Based on our clinical finding and with the diagnosis of B12 deficiency we started B12 1000mg/d for the patient. For ocular problems, eye drop of chloramphenicol and lubricants were described. After 3 days the patient complained of foreign body sensation, which was an indication of corneal sensation improvement and after 3 weeks he showed a dramatic response with corneal sensation reversal, increase of visual acuity, improved neurotrophic keratopathy and significantly improved optic neuropathy and neurological findings Fig. (**6a,b**) . The blood B12 level at this point increased to 300 pgr/ml, well into the normal range.

## 3 DISCUSSION

B12 deficiency is a relatively rare nutritional problem that might occur second to malnutrition or malabsorption. It is usually due to inadequate absorption associated with Pernicious Anemia (PA) or secondary to gastric disease like autoantibody formation, chronic atrophic gastritis, gastrectomy and gastritis intestinal disorders [4].

 Also, drugs usage like prolonged use of proton pump inhibitors (*e.g*, omeprazole) as well as inadequate dietary intake like in strict vegetarians can cause this condition [5]. In addition, women who are only moderate vegetarians may become B12 deficient during pregnancy and lactation and their infants may also be deficient [6]. Other probable causes are HIV infection, nitrous oxide exposure and hereditary causes like the production of a qualitatively abnormal intrinsic factor or decreased uptake of the intrinsic factor-cobalamin complex [4].

 In our case, we confronted two ophthalmological problems: the first was neurotrophic keratopathy and the second was optic neuropathy. The patient had some neurological problems like ataxia, limbal parasthesia and lethargy. Based on this problem list we suspected vitamin B12 deficiency. In addition, para clinic findings showed positive points like spinal MRI with hyper intensity in the posterior column, hyper homocysteinemia, iron deficiency and normocytic anemia. Lower than normal levels of blood B12 were detected and after starting 1000mg/d B12 for the patient, his dramatic response regarding the neurologic signs and keratopathy after 3 weeks confirmed our diagnosis.

## CONCLUSION

In conclusion based on our findings, it seems that B12 deficiency might be a very rare cause of neurotrophic keratopathy.

## Figures and Tables

**Fig. (1):**
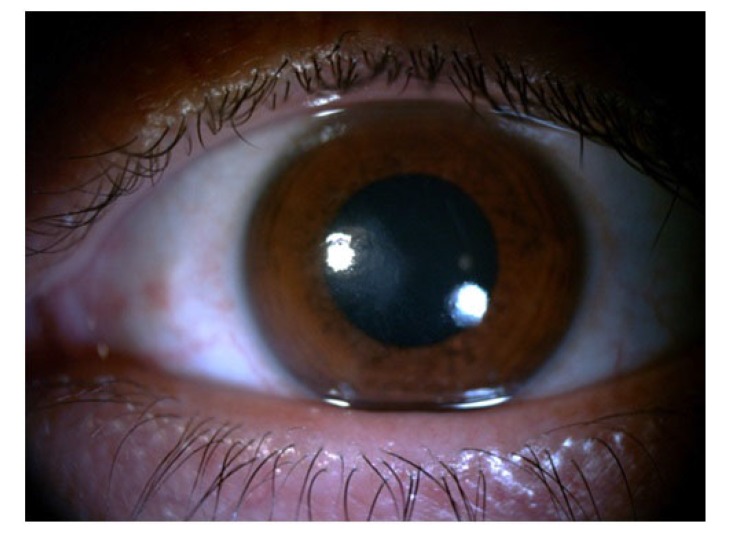
Corneal sub epithelial infiltration without any foreign body sensation due to decreased corneal sensation.

**Fig. (2):**
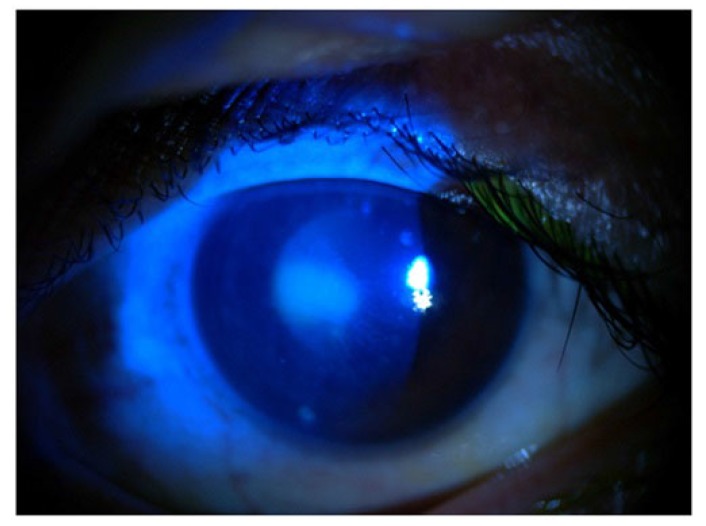
Corneal epithelial defect and abrasion observed after fluorescein staining.

**Fig. (3):**
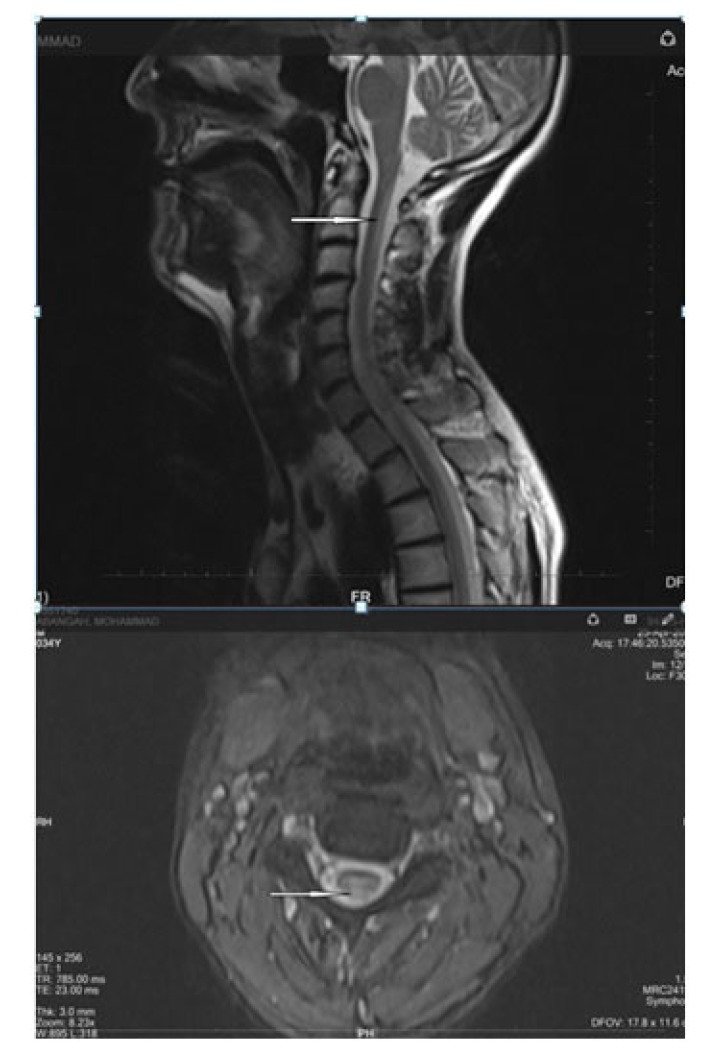
Hyperintensity in posterior column observed in MRI (arrows).

**Fig. (4a,b):**
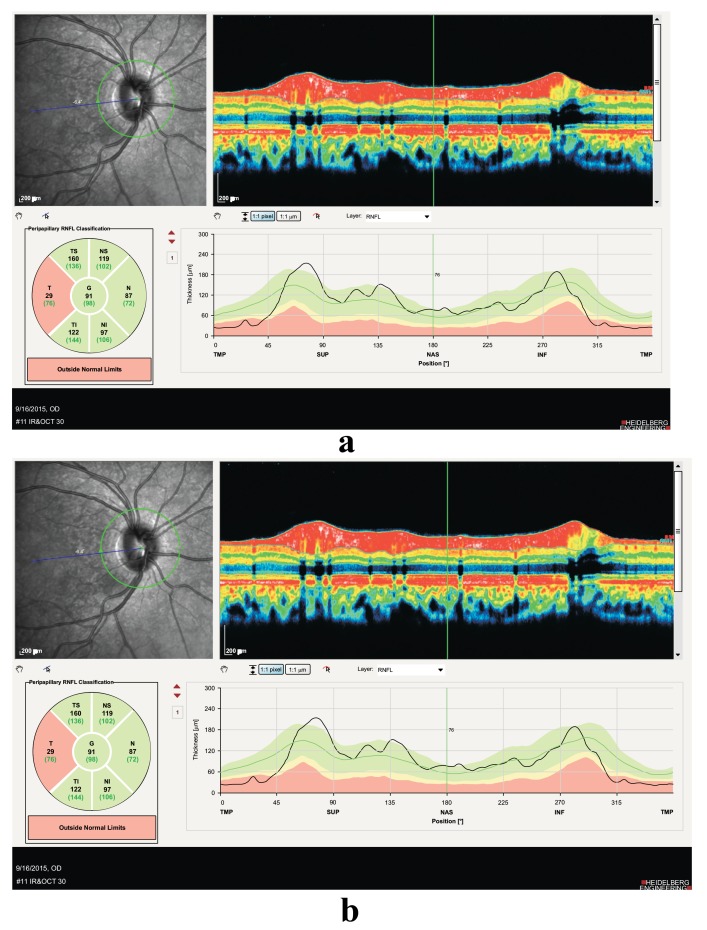
Optic atrophy in temporal zones observed in OTC in right eye (4a) and the left Eye (4b).

**Fig. (5a,b):**
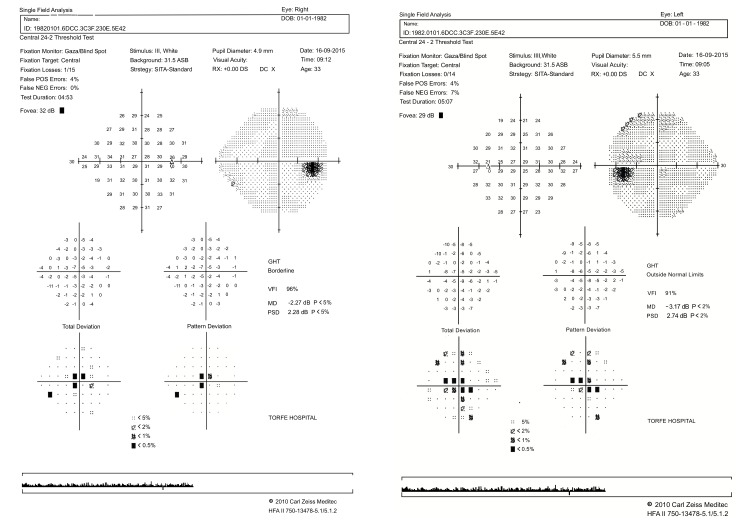
Bilateral central scotoma in perimetry of right eye (5a) and the left eye (5b).

**Fig. (6a,b):**
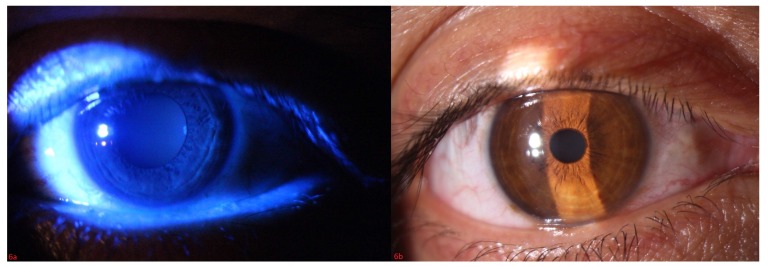
The corneal healing and resolve of corneal abrasion observed after treatment with B12.
